# spyrmsd: symmetry-corrected RMSD calculations in Python

**DOI:** 10.1186/s13321-020-00455-2

**Published:** 2020-08-31

**Authors:** Rocco Meli, Philip C. Biggin

**Affiliations:** Department of Biochemistry, South Parks Rd., Oxford, OX1 3QU UK

**Keywords:** RMSD, Symmetry, Software, Python

## Abstract

Root mean square displacement (RMSD) calculations play a fundamental role in the comparison of different conformers of the same ligand. This is particularly important in the evaluation of protein-ligand docking, where different ligand poses are generated by docking software and their quality is usually assessed by RMSD calculations. Unfortunately, many RMSD calculation tools do not take into account the symmetry of the molecule, remain difficult to integrate flawlessly in cheminformatics and machine learning pipelines—which are often written in Python—or are shipped within large code bases. Here we present a new open-source RMSD calculation tool written in Python, designed to be extremely lightweight and easy to integrate into existing software.

## Introduction

Computational structure-based drug discovery has steadily gained traction partially thanks to the constant improvements in available software, now often free and open source. Protein-ligand docking in particular is now a standard tool employed in the early stages of drug discovery pipelines in order to screen possible drugs acting on a known target of interest.

Protein-ligand docking consists of the prediction of binding modes and binding affinity of a (flexible) ligand to a target of known structure. The performance of docking programs is often assessed by their ability to reproduce the crystallographic pose of the bound ligand. A common metric to evaluate the difference between the predicted binding pose and the crystallographic pose is the heavy-atoms root mean square displacement (RMSD) [1], although other metrics have been suggested [2]. RMSD calculations are also used in other contexts, for example for the evaluation of diversity in generated conformers [3].

Many simple scripts to compute RMSDs are based on the assumption of a direct one-to-one mapping between atoms of different conformers of the same ligand. In different words, atoms are often assumed to be labelled according to their position in a coordinate file (or data structure) and they are paired according to such label. This assumption breaks down when such labels are not conserved—i.e. the order of atoms is different in the two structures being compared—and/or for symmetric molecules. In the case of symmetric molecules, different binding poses can be chemically identical but different in terms of atom-atom mapping. Since molecular connectivity is naturally represented by graphs (atoms as vertices and bonds as edges), tools from graph theory can be used to obtain the correct atom-atom mapping for two different conformers of the same molecule, thus avoiding the problems outlined above.

Here we present a new Python tool, spyrmsd, for the calculation of symmetry-corrected RMSDs based on graph isomorphisms.

## Implementation

spyrmsd is implemented in pure Python and therefore it is easy to integrate in existing Python libraries and Python pipelines, particularly common in cheminformatics and machine learning projects. In this section we describe the implementation of the different types of RMSD calculations implemented in spyrmsd, their use, and their shortcomings.

### Standard RMSD

Let us call $\mathbf {A}$ and $\mathbf {B}$ the $N\times 3$ matrices of atomic coordinates of two conformers A and B of the same molecule. The standard RMSD is simply defined as

$$\begin{aligned} \text {RMSD}_\text {standard} = \sqrt{\frac{1}{N}\sum _{i=1}^{N}\sum _{j=0}^2 (A_{ij} - B_{ij})^2 }. \end{aligned}$$

If we define the displacement $\mathbf {r}_i = \mathbf {a}_i - \mathbf {b}_i$—where $\mathbf {a}_i$ is the *i*-th row of $\mathbf {A}$ and $\mathbf {b}_i$ is the *i*-th row of $\mathbf {B}$—the standard RMSD can be written more compactly as

1 $$\begin{aligned} \text {RMSD}_\text {standard} = \sqrt{\frac{1}{N}\sum _{i=1}^{N}\mathbf {r}_i^2}. \end{aligned}$$

This simple formula, which assumes the atomic coordinates to be provided in the same order for both conformers, is easy to compute. In spyrmsd the calculation of $\text {RMSD}_\text {standard}$ is vectorised using numpy [4] for speed.

**Fig. 1:**
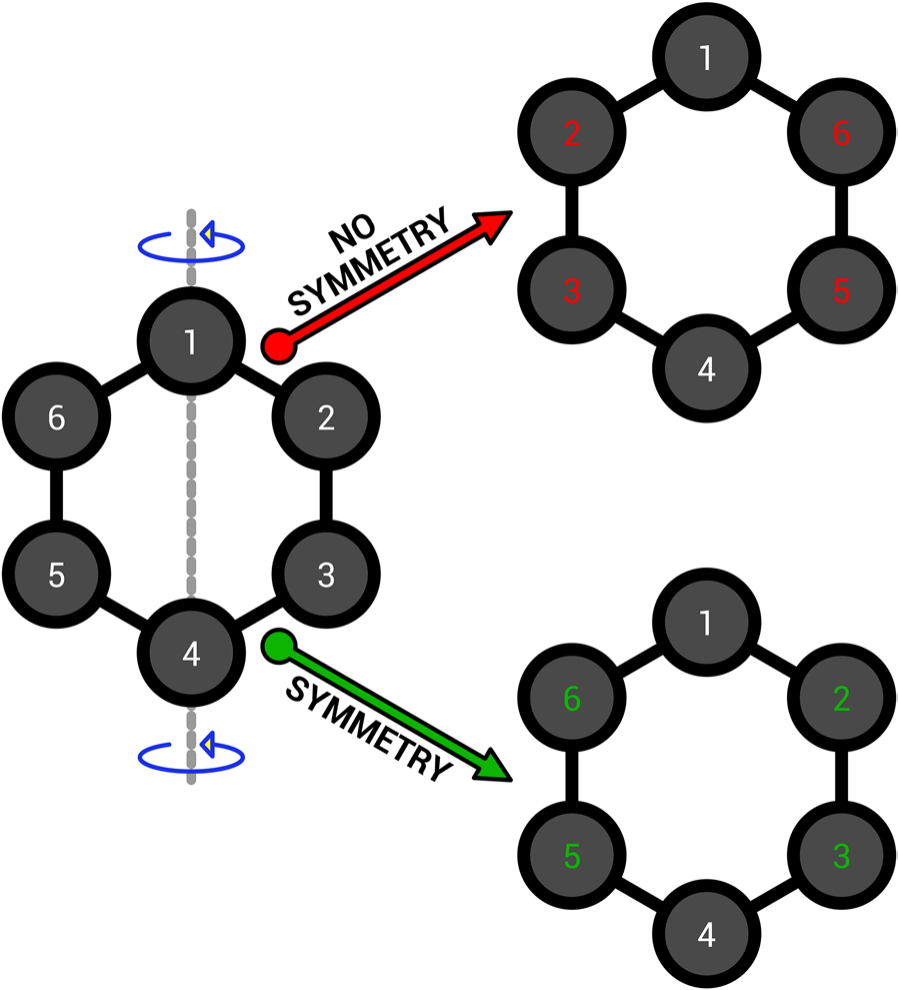
Atom–atom mapping for the benzene molecule after a mirror operation with and without symmetry correction

A serious drawback of standard RMSD calculations is that they do not take into account molecular symmetry. This is problematic since atoms of the same specie are intrinsically indistinguishable and therefore symmetry operations conserve molecular properties. Figure 1 shows the atom–atom mapping for benzene with and without symmetry correction after a mirror operation; it is clear that a simple positional atom–atom mapping leads to artificially inflated results. Symmetry corrections would lead to the correct result expected with indistinguishable atoms.

### Quaternion characteristic polynomial method

Standard RMSD calculations take into account the possible translations between the two conformers. In order to measure conformational similarity—and neglect translations—the RMSD can be computed on optimally superimposed structures. This minimised RMSD for a pair of molecules can be computed efficiently using the quaternion characteristic polynomial (QCP) method [5], which circumvent the need of finding orthogonal (rigid-body) rotations and special considerations for edge cases.

The QCP methods is based on the calculation a $4\times 4$ symmetric key matrix [5]

$$\begin{aligned} \mathbf {K} = \begin{pmatrix} S_{00}+S_{11}+S_{22} &{} S_{12}-S_{21} &{} S_{20}-S_{02} &{} S_{01}-S_{10} \\ &{} S_{00}-S_{11}-S_{22} &{} S_{01}+S_{10} &{} S_{20}+S_{02} \\ &{} &{} -S_{00}+S_{11}-S_{22} &{} S_{12}+S_{21} \\ &{} &{} &{} -S_{00}-S_{11}+S_{22} \\ \end{pmatrix} \end{aligned}$$

where

$$\begin{aligned} S_{ij} = \sum _{k=0}^{N-1} B_{ki}A_{kj}. \end{aligned}$$

The minimum RMSD is then given by [5]

2 $$\begin{aligned} \text {RMSD}_\text {min} = \sqrt{\frac{G_A + G_B - 2\lambda _\text {max}}{N}} \end{aligned}$$

where $G_A = {{\,\mathrm{Tr}\,}}{\mathbf {A}^T \mathbf {A}}$, $G_B = {{\,\mathrm{Tr}\,}}{\mathbf {B}^T \mathbf {B}}$ and $\lambda _\text {max}$ is the maximum eigenvalue of $\mathbf {K}$. The eigenvalues of $\mathbf {K}$ can be obtained by finding the roots of the characteristic polynomial $P(\lambda ) = \det \left( \mathbf {K} - \lambda \mathbf {I}\right)$ [6], where $\mathbf {I}$ is the identity matrix. For the matrix $\mathbf {K}$ the characteristic polynomial is given by [5]

$$\begin{aligned} P(\lambda ) = \lambda ^4 + C_2 \lambda ^2 + C_1 \lambda + C_0, \end{aligned}$$

where $C_2 = -2{{\,\mathrm{Tr}\,}}{\mathbf {M}^T\mathbf {M}}$ (with $\mathbf {M} = \mathbf {B}^T\mathbf {A}$), $C_1 = -8\det {\mathbf {M}}$ and $C_0 = \det {\mathbf {K}}$. The largest characteristic polynomial root $P(\lambda _\text {max}) = 0$ can be efficiently computed using the Newton-Raphson method [7] starting from the initial guess $(G_A + G_B) / 2$ [5].

Care should be taken when the two molecules A and B overlap perfectly. In such case, $\lambda _\text {max} = (G_A + G_B) / 2$ and therefore the term $G_A + G_B - 2\lambda _\text {max}$ in Eq. (2) can become negative due to numerical errors.

In spyrmsd the solution of the characteristic polynomial equation $P(\lambda _\text {max}) = 0$ is based on the Newton–Raphson method implemented in scipy [8] while other vector and matrix operations are vectorised using numpy.

### Hungarian algorithm for symmetry correction

The Hungarian algorithm [9, 10] is an algorithm to solve the linear sum assignment problem [11] (also known as minimum weight matching in bipartite graphs) and has been previously proposed as a method to introduce symmetry corrections in RMSD calculations [12]. If $\mathbf {D}$ is the $N\times N$ matrix of squared pairwise distances between all atoms of the conformer A to all atoms of the conformer B, the linear weight assignment problem consists in finding the assignment matrix $\mathbf {X}$ that minimises the assignment cost $\sum _{ij} D_{ij}X_{ij}$, where $X_{ij} = 1$ if and only if atom *i* of conformer A is assigned to atom *j* of conformer B. The RMSD computed using the Hungarian algorithm is therefore given by

$$\begin{aligned} \sqrt{\frac{1}{N}\min _\mathbf {X} \sum _{ij} D_{ij}X_{ij}}, \end{aligned}$$

under the constraint that each row is assigned to exactly one column and each column to exactly one row. This definition is however problematic, since the solution of the assignment problem could end up pairing atoms of different elements. In order to avoid this drawback, the assignment problem is solved for every element separately [12]. If $\mathbf {D}^e$ is the $N_e\times N_e$ matrix of squared pairwise distances between atoms of element *e* of conformer A to atoms of element *e* of conformer B, the RMSD computed using the Hungarian algorithm is given by

$$\begin{aligned} \text {RMSD}_\text {Hungarian} = \sqrt{\frac{1}{N}\sum _e \min _{\mathbf {X}^e}\sum _{ij} D^e_{ij} X^e_{ij}}, \end{aligned}$$

where $X^e_{ij} = 1$ if and only if atom *i* of element *e* in conformer A is assigned to atom *j* of element *e* in conformer B and were the sum on *e* runs over all elements of the molecule.

**Fig. 2:**
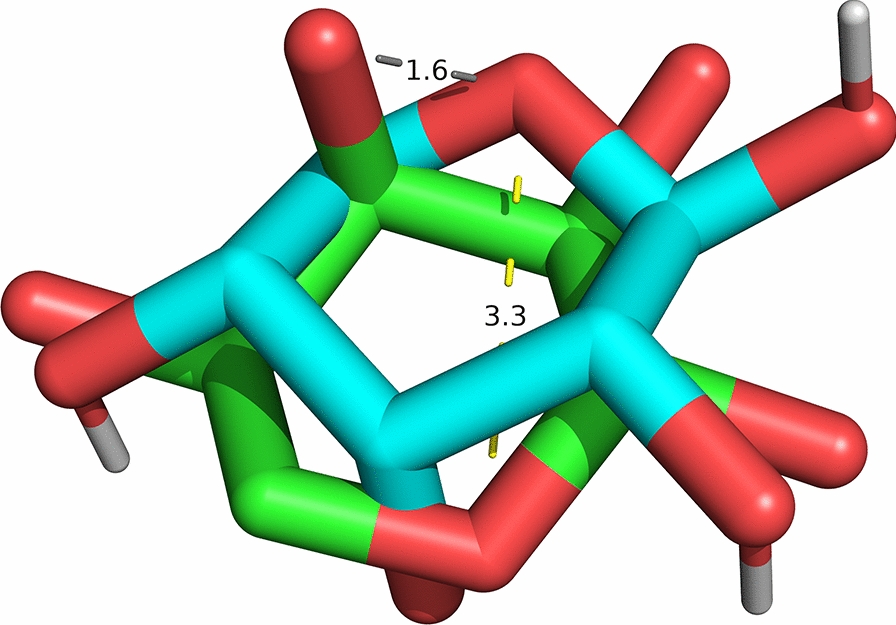
Crystal pose (green) and second-best docking pose (cyan) for the ligand of the protein-ligand complex 1DRJ. The Hungarian method assigns the ring oxygen to an oxygen atom nearby ($d = 1.6\,{\AA }$, grey) while the graph isomorphism method correctly maps one ring oxygen to the other ($d = 3.3\,{\AA }$, yellow). The Hungarian method results in an artificially low RMSD of 1.00 Å, compared to the correct RMSD of 2.46 Å obtained with graph isomorphisms

Even when the Hungarian algorithm is used to assign atoms of the same element, problems can arise from the fact that the algorithm is not aware of the overall molecular structure. This could result in unphysical assignments which break the molecular graph and result in artificially low RMSD values [13]. Figure 2 shows a simple situation where unphysical assignments arise and lead to a RMSD value lower than the correct one.

### Graph isomorphisms for symmetry correction

In order to overcome the problems of the Hungarian algorithm, tools from graph theory can be borrowed to perform an optimal atom-atom assignment based on graph isomorphisms. A molecule can be represented as a graph ${\mathcal {G}}(V,E)$—hereafter referred to molecular graph—where the vertices *V* are associated to atoms and the edges *E* are associated to bonds. If two conformers A and B are represented by graphs ${\mathcal {G}}_\text {A}$ and ${\mathcal {G}}_\text {B}$, respectively, the mapping of atoms of molecule A to atoms of molecule B becomes a graph isomorphism problem. An isomorphism between graphs ${\mathcal {G}}_\text {A}$ and ${\mathcal {G}}_\text {B}$ is a bijective mapping of the vertices of graph A to vertices of graph B that preserves the edge structure of the graphs (molecular connectivity in the case of molecular graphs). With the bijective mapping connecting vertices (atoms) of ${\mathcal {G}}_\text {A}$ to vertices (atoms) of ${\mathcal {G}}_\text {B}$ the RMSD between the two molecules can be computed using the standard RMSD formulation of Eq. (1).

spyrmsd can leverage networkx [14] or graph-tool [15] for graph representation and graph matching. All possible graph isomorphisms are computed using the VF2 algorithm [16] and the lowest RMSD among all isomorphisms is retained.

The graph isomorphism problem is a non-polynomial (NP) problem and therefore symmetry-corrected RMSD calculations are only suited for small to medium sized molecules. In order to improve speed, graph isomorphisms are cached by default when computing the RMSD between multiple conformations of the same molecule.

### API

The main module of spyrmsd is the rmsd module, where all the high-level RMSD functions are implemented. The following functions are available to the user:

- rmsd for the computation of the standard RMSD,
- hrmsd for the computation of RMSD using the Hungarian algorithm,
- symmrmsd for the computation of symmetry-corrected RMSD,

symmrmsd should always be used for small molecules, in order to get the right symmetry-corrected RMSD. rmsd is provided to compute the standard RMSD when symmetry does not play a role (or when the molecular graph is too large to efficiently apply symmetry-corrections) and atoms are listed in the same order. hrmsd is provided for comparison with existing implementations and should not be used otherwise, because of the problems outlined above.

The minimum RMSD (computed using the QCP method [5]) can be obtained with the keyword minimize=True, with and without symmetry-corrections.

spyrmsd is designed to be easily integrated in existing Python libraries or pipelines. For this reason the application programming interface (API) is minimalistic: only atomic coordinates and atomic numbers (rmsd and hrmsd), and molecular adjacency matrices (symmrmsd) have to be passed to RMSD functions in the form of numpy arrays. This simple API makes spyrmsd completely agnostic of the way molecules are stored in different software, as long as they can provide the minimal information required.

### Standalone RMSD tool

spyrmsd also offers a standalone RMSD tool as a command line interface (CLI) exposing the functionality of the rmsd and symmrmsd functions. The hrmsd function is not exposed in the CLI, to avoid erroneous calculations.

In the standalone tool, molecular input is handled by OpenBabel [17] (via its Python interface pybel [18]) or RDKit [19]. Such packages are also responsible for building the adjacency matrix representing the molecular graph.

OpenBabel’s own RMSD calculation tool, obrms, is expected to be faster than spyrmsd as a standalone tool since it does not have any Python overhead.

## Results

### Testing

In order to test the correctness of spyrmsd against OpenBabel’s obrms we redocked the PDBbind refined set [20, 21] with smina [22] to generate different ligand conformations. Ligand SDF files were downloaded directly from the PDB [23] in order to avoid problems with the connectivity present in the original PDBbind dataset. smina was run using the default settings with protein PDB files stripped of water molecules. The top 10 binding poses were retained, resulting in a total of 40,439 different conformations. The RMSD of each docked pose with respect to the crystal pose was computed using symmetry-corrected RMSD with and without minimisation (using the QCP method [5]).

**Fig. 3:**
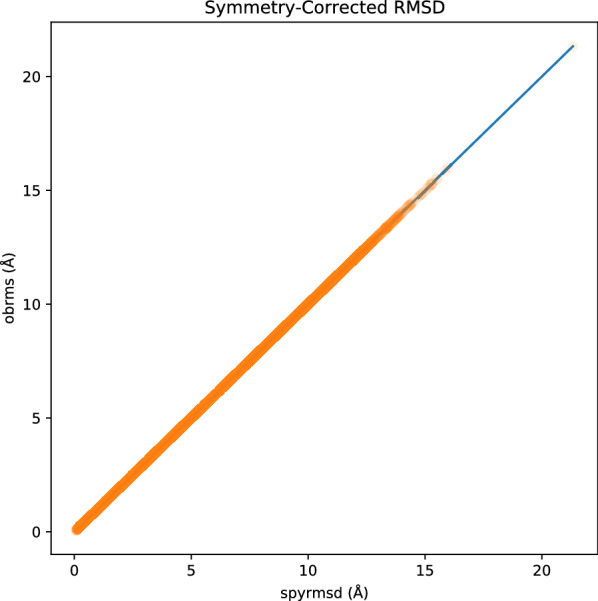
Comparison between obrms and spyrmsd for symmetry-corrected RMSD calculations. The mean squared error is $3.80\times 10^{-11}$ while the Pearson’s correlation coefficient is 1.00. The maximum absolute error is $5.00\times 10^{-5}$ amongst all 40439 system tested

**Fig. 4:**
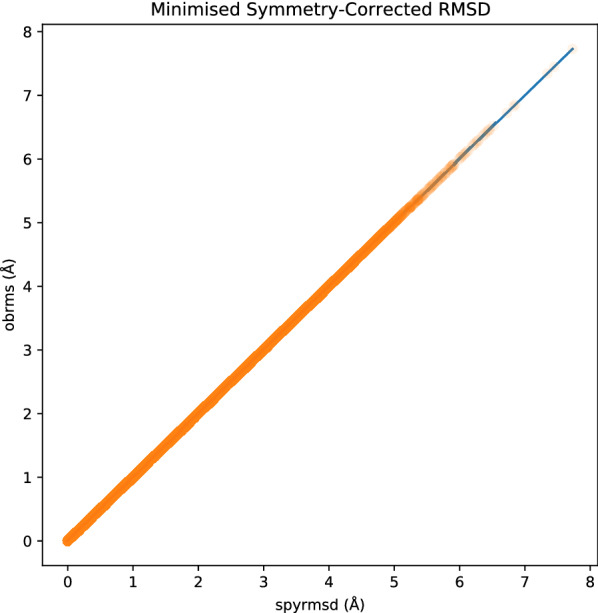
Comparison between obrms and spyrmsd for minimised symmetry-corrected RMSD calculations. The mean squared error is $3.28\times 10^{-12}$ while the Pearson’s correlation coefficient is 1.00. The maximum absolute error is $5.00\times 10^{-6}$ amongst all 40439 system tested

Figures 3 and 4 show the relationship between RMSDs obtained with spyrmsd and obrms with and without minimisation. The RMSDs computed with the two softwares correlates perfectly (Spearman’s correlation coefficient of 1.00) and present a maximum absolute error of $5\times 10^{-5}{{\AA }}$. This gives us great confidence that the two independent implementations are equivalent (Additional file 1).

**Fig. 5:**
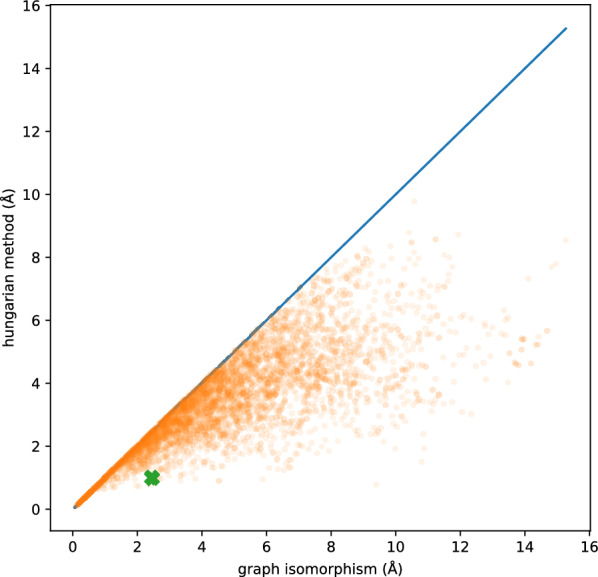
Comparison between symmetry-correction performed with the Hungarian method or leveraging graph isomorphisms. The Hungarian algorithm often results in artificially low RMSDs due to atom-atom assignments breaking the molecular connectivity. The green cross corresponds to the protein-ligand complex 1DRJ analysed in Fig. 2

A comparison between the Hungarian method and symmetry-corrected RMSD obtained via graph isomorphisms is presented in Fig. 5. As previously pointed out, the Hungarian method can result in assignments incompatible with the molecular connectivity and therefore leads to artificially low RMSD values [13]. Therefore, the hrmsd function is provided only for comparison with existing software and should not be used otherwise.

### Speed

By design, spyrmsd is written fully in Python and leverages fast libraries that are easy to install (using the pip or conda package managers). This means that there is some overhead compared to the most efficient implementations in other compiled libraries.

**Fig. 6:**
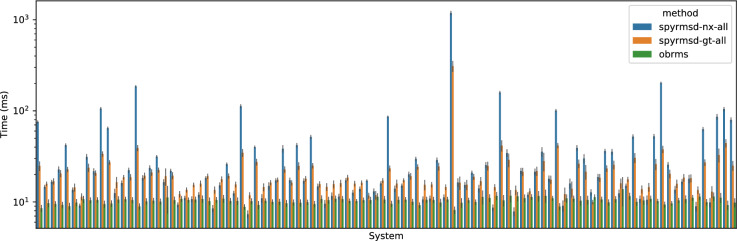
RMSD calculation time (including input) for 100 randomly selected systems. Error bars indicate the standard deviation over 25 repeats. spyrmsd is comparable or an order of magnitude slower than obrms

**Fig. 7:**
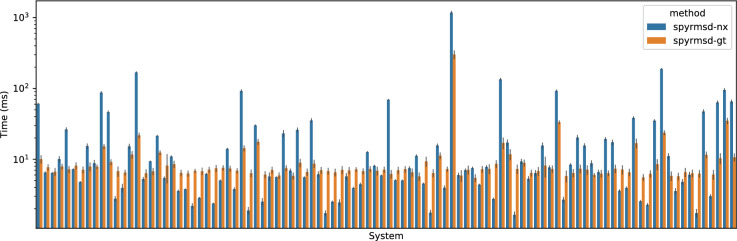
RMSD calculation time (without input) for 100 randomly selected systems. Error bars indicate the standard deviation over 25 repeats. networkx shows a large variability between systems, while graph-tool is more consistent

Figure 6 shows a speed comparison between spyrmsd and obrms for 100 randomly selected systems. Error bars are obtained by repeating the measurements 25 times. spyrmsd is usually comparable or an order of magnitude slower than obrms. This is expected since Python comes with some overhead compared to compiled code. The difference between the graph-tool and networkx backends is more difficult to elucidate: graph-tool seems to be generally slightly faster, but networkx has clearly more variation from system to system (see Fig. 7).

Benchmarking was performed on an Apple MacBook Pro (macOS 10.15) with a 2.6 GHz 6-Core Intel Core i7 processor and 32 GB of 2400 MHz DDR4 memory (Additional file 2).

## Discussion

Despite being somewhat slower than other state-of-the-art tools for RMSD calculation, we believe that spyrmsd could be extremely useful to the community: it is a lightweight tool with focussed functionality, it is easy to use and integrate in existing Python codebases and pipelines, and it is easy to install via popular package managers.

### Easy installation

spyrmsd is available on the Python Package Index (PyPI) [24] and via the conda package manager [25] on the conda-forge channel [26]. This provides easy cross-platform installation of spyrmsd and all its dependencies to work as a library (with networkx). On macOS and Linux, users can get some speed improvement by installing graph-tool, which is also available via the conda package manager.

In order to use spyrmsd as a standalone tool, users will have to install either OpenBabel or RDKit with their preferred installation method.

### Easy integration in existing libraries

spyrmsd is easy to integrate into existing pipelines thanks to its clean and simple API. Standard RMSD calculations require atomic coordinates and atomic numbers only, while symmetry-corrected RMSD calculations also require adjacency matrices in order to compute graph isomorphisms. Atomic coordinates and atomic numbers are usually readily available in most Python libraries dealing with molecular file formats, while the adjacency matrix of a molecule is easy to build from bond connectivity.

We believe that the simple API will favour the integration of spyrmsd in many existing libraries, bringing symmetry-corrected RMSD calculations to widely used packages.

### Software best practices

The development of spyrmsd is based on modern software engineering best practices. The code is version-controlled using git [27] and it is freely available on GitHub (https://github.com/RMeli/spyrmsd) [28], released under the open-source and permissive MIT license.

The code is extensively tested using pytest [29]. Tests are run automatically every time a new version of the code is pushed to GitHub thanks to Travis-CI bindings for continuous integration [30]. The code coverage of the test suite is reported on Codecov [31], which provides easy-to-read reports. A code coverage of 100% is targeted, so that all lines of code are executed at least once during tests.

The code is compatible with Python 3.6 or above. Static analysis tools are constantly applied to the code in order to catch errors that would be otherwise missed or discovered only during execution. We use mypy to perform static checks [32] and flake8 to detect style and formatting issues. Such tools help maintaining correctness and stability for future developments as well as a clean codebase.

Finally, the code is documented using Python docstrings and the documentation is built automatically using sphinx [33]. This will likely make it easier to fully understand the codebase thus facilitating the adoption of spyrmsd by other libraries.

## Conclusion

spyrmsd provides robust symmetry-corrected RMSD calculations with a clean and simple API that is easy to integrate in existing Python libraries and pipelines. We believe that such a tool could be useful to the wider community of molecular modellers and cheminformaticians.

Future development of the software will focus on improved automatic bond perception (to automatically build molecular adjacency matrices) and speed.

## Availability and requirements

- Project name: spyrmsd
- Code: https://github.com/RMeli/pyrmsd
- Docs: https://spyrmsd.readthedocs.io/
- Operating systems: Linux, macOS, Windows
- Programming language: Python
- Other requirements: Python 3.6 or higher
- License: MIT

## Supplementary information

**Additional file 1.** Comparison of correctness between spyrmsd and obrms.

**Additional file 2.** Speed comparison between spyrmsd (with networkx or graph-tool) and obrms.

## Data Availability

spyrmsd is available for download on PyPI and conda-forge, while the source code is available on GitHub under the MIT license: https://github.com/RMeli/pyrmsd. The code used to produce the figures is included in the supplementary information. The results of docking are available on Zenodo (https://doi.org/10.5281/zenodo.3747315).
